# Assessing Patient Experience and Healthcare Quality of Dental Care Using Patient Online Reviews in the United States: Mixed Methods Study

**DOI:** 10.2196/18652

**Published:** 2020-07-07

**Authors:** Ye Lin, Y Alicia Hong, Bradley S Henson, Robert D Stevenson, Simon Hong, Tianchu Lyu, Chen Liang

**Affiliations:** 1 College of Dental Medicine Western University of Health Sciences Pomona, CA United States; 2 College of Health and Human Services George Mason University Fairfax, VA United States; 3 Arnold School of Public Health University of South Carolina Columbia, SC United States

**Keywords:** dental care, healthcare quality, consumer health informatics, patient online reviews, patient review websites, natural language processing

## Abstract

**Background:**

Over the last two decades, patient review websites have emerged as an essential online platform for doctor ratings and reviews. Recent studies suggested the significance of such websites as a data source for patients to choose doctors for healthcare providers to learn and improve from patient feedback and to foster a culture of trust and transparency between patients and healthcare providers. However, as compared to other medical specialties, studies of online patient reviews that focus on dentists in the United States remain absent.

**Objective:**

This study sought to understand to what extent online patient reviews can provide performance feedbacks that reflect dental care quality and patient experience.

**Methods:**

Using mixed informatics methods incorporating statistics, natural language processing, and domain expert evaluation, we analyzed the online patient reviews of 204,751 dentists extracted from HealthGrades with two specific aims. First, we examined the associations between patient ratings and a variety of dentist characteristics. Second, we identified topics from patient reviews that can be mapped to the national assessment of dental patient experience measured by the Patient Experience Measures from the Consumer Assessment of Healthcare Providers and Systems (CAHPS) Dental Plan Survey.

**Results:**

Higher ratings were associated with female dentists (*t*_71881_=2.45, *P*<.01, *g*=0.01), dentists at a younger age (*F*_7, 107128_=246.97, *P*<.001, *g*=0.11), and those whose patients experienced a short wait time (*F*_4, 150055_=10417.77, *P*<0.001, *g*=0.18). We also identified several topics that corresponded to CAHPS measures, including discomfort (eg, painful/painless root canal or deep cleaning), and ethics (eg, high-pressure sales, and unnecessary dental work).

**Conclusions:**

These findings suggest that online patient reviews could be used as a data source for understanding the patient experience and healthcare quality in dentistry.

## Introduction

Over the last few years, patient review websites have gained increasing interest among health consumers, academic communities, and healthcare providers [1,2]. A tremendous amount of online patient reviews were shared with the public through patient review websites, becoming a common source of information for patients choosing a doctor. Accordingly, a growing body of literature in online patient review studies has been developed using public health informatics methods to leverage the distribution and determinants of data for informing public health and public policy, coinciding with the Research Framework of Infodemiology and Infoveillance [3].

Despite debates on whether patient-generated reviews would be useful to improve healthcare quality [4-6], online reviews could serve as a valuable data source for understanding patient experience and the patient-provider relationship, and present unique values in improving dental care. Online reviews play an increasingly important role in health consumers’ decision-making for choosing dental services [7,8]. A recent national survey reported that 59% of respondents recognized the importance of patient review websites, 35% selected a doctor based on good ratings, and 37% avoided selecting a doctor based on bad ratings. Nearly 90% of the respondents rated listings of accepted health insurance on review sites as high importance [2]. Online patient review studies also found strong associations between ratings and doctor characteristics, such as gender, age, years of practice, online presence, and medical education and training, with variations at specialties [1,9].

Online reviews are also rich in data on the patient experience, a critical measure of healthcare quality [10]. The notion of patient experience includes the entire scope of interactions, from appointment scheduling and access to information to communications with clinicians, cost, and payment. Positive patient experience is associated with better prevention and treatment adherence, safer healthcare outcomes, and better utilization of healthcare resources [11-15]. Incorporating data from the patient experience is also consistent with the principles of patient-centered care [10], as well as the goal of public reporting and the performance-based payment model set by the Centers for Medicare and Medicaid Services (CMS) [16].

Online patient reviews can inform better decision-making among patients and can be used to improve healthcare quality. As such, many studies on online patient reviews in a variety of medical specialties have been reported [1]. Nevertheless, analyses of online reviews of dentists are sparse. We only identified one study of online patient reviews in dentistry conducted in Germany [17]. In the United States, studies of dental patient reviews are missing. To address the literature gaps, we conducted a study with two aims. The first aim was to characterize the online reviews of dental patients in the United States by analyzing data extracted from patient review websites. In particular, we examined the association between dentist characteristics and patient ratings.

Our second aim was to understand to what extent online reviews can inform assessments of the patient experience by identifying semantic mentions of the patient experience in patient reviews. Dental patient experience is traditionally assessed with the CAHPS (Consumer Assessment of Healthcare Providers and Systems) Dental Plan Survey, administered by CMS [18]. Using online reviews for patient experience assessment is feasible but challenging. For instance, information extraction from online patient reviews is labor-intensive because the reviews are written in free text. Content analysis frequently used in social science research could not be applied to this study, in which there were hundreds of thousands of reviews to assess. The identification of information relevant to the patient experience is also necessary to ensure research validity, yet there are no guidelines or empirical studies on how to identify patient experience content in online reviews. Patient reviews cover a variety of topics, but not all of them are related to the patient experience. For example, topics of patient satisfaction should not be identified as patient experience. The patient experience is comprised of the clinical encounters and has been frequently used as an indicator of patient safety [19], whereas patient satisfaction relates to whether patients’ expectations about healthcare are met [20]. To overcome these challenges, we incorporated natural language processing [21] and human expert evaluation to identify relevant topics from online patient reviews. Natural language processing has extensive applications in health informatics and medical internet research, in which it has vastly improved the efficiency of processing free-text data using advanced statistical methods and automated computing [3]. Human expert evaluation mitigates the problem of misprocessed data typically produced by such a computerized method and improves the interoperability of the data analysis and results.

## Methods

### Data Acquisition

Publicly accessible online review data were obtained from HealthGrades, a well-known patient review website in the United States. Among many other sites, we focused on HealthGrades for two reasons. First, HealthGrades is widely used by patients who receive healthcare services from a full range of medical specialties in the United States. Second, HealthGrades provides a well-organized sitemap structure that facilitates data extraction. We analyzed data from a single review site because the data structure and measures of dentist demographics and performance vary, which will hinder data consolidation. In addition, data from multiple patient review sites would have little impact on representation and generalizability of this study because an active dentist typically has profiles on all popular patient review sites.

Online reviews for 204,751 dentists were extracted. This census approximates but does not fully match the workforce statistics (199,486 working dentists as of 2018) reported by the American Dental Association (ADA) [22] because some profiles are for recently inactive dentists and misclaimed or inappropriately captured profiles. The data contain the following attributes: state, city, specialty, gender, age, language, education, number of reviews, ratings, reviews, and wait time.

### Data Preprocessing

There were 41 dental service listings in HealthGrades. We categorized these services into the 10 dental specialties defined by the ADA, general dentistry, and others (ie, unidentified and miscellaneous), resulting in 8 specialties (ie, dental anesthesiology, endodontics, oral and maxillofacial pathology, oral and maxillofacial surgery, orthodontics and dentofacial orthopedics, pediatric dentistry, periodontics, and prosthodontics), general dentistry, and others for downstream analyses. Public health dentistry and oral and maxillofacial radiology were excluded as they have only one entry at most. The data extraction was completed in September 2019. The study was identified as a nonhuman study by the Institutional Review Board of the University of South Carolina.

### Statistical Analyses

We employed statistical analyses to assess the associations between ratings and dentists’ characteristics using R Project for Statistical Computing. We used descriptive statistics to calculate proportions and mean distributions. Based on reported online reviews studies in general medical specialties, we hypothesized that (1) female dentists, (2) young dentists, and (3) short wait times would be associated with higher overall ratings. We also hypothesized that specialties are associated with overall ratings. An independent sample t-test was used to test whether ratings differ by gender. Analysis of Variance (ANOVA) was used to test whether ratings differ by specialties, age, and wait time, respectively. We used Hedges *g* to approximate the effect size because of unbalanced sample sizes in comparison groups.

### Text Mining

**Figure 1:**
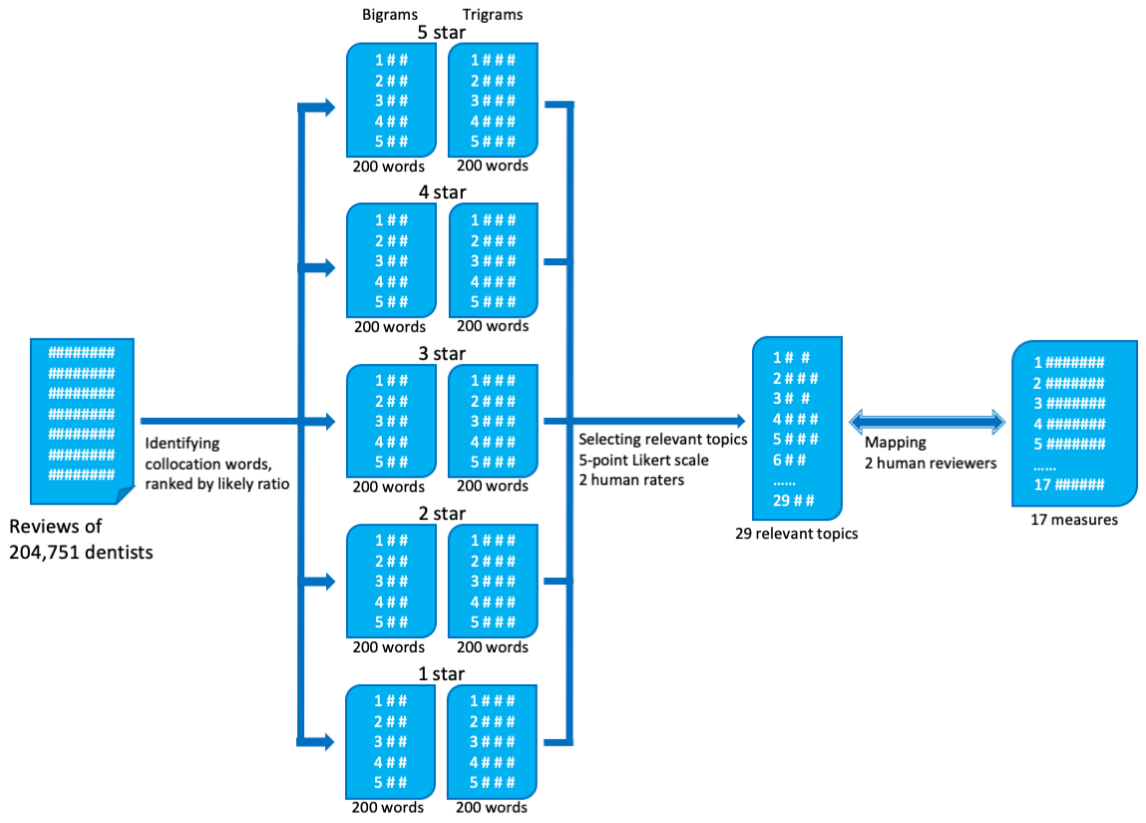
Diagram of text mining procedures.

Semi-automated natural language processing was used to identify concepts related to patient experience with limited human labor required. Figure 1 is a diagram of text mining procedures. We used Python for text processing and computation. Built on our pilot study of analyzing patient-generated reviews [23], we first extracted reviews from online sites, followed by standard data cleaning procedures, including tokenization and removal of stop words.

We then calculated bigram and trigram collocations. Collocations are habitual expressions of multiple words. In this study, we ranked using the “likelihood ratio” method [24], top 200 bigram and trigram collocations, respectively, from reviews associated with every rating category (ie, 1, 2, 3, 4, 5).

We observed a number of the collocations that are irrelevant to concepts of dental care and patient experience (eg, “phone call” and “many years ago”) but were still ranked top 200 by likelihood ratio. Therefore, two raters (YL and CL) independently picked collocations related to patient experience using a 4-point Likert scale (“definitely relevant,” “somewhat relevant,” “somewhat irrelevant,” “definitely irrelevant”). Inter-rater reliability was assessed using Cohen kappa. Two raters discussed on the collocations that received contrary opinions (relevant vs irrelevant) until a consensus was reached.

Next, we mapped patient-experience-related concepts onto a total of 17 composite measures from the Patient Experience Measures from the CAHPS Dental Plan Survey. Table 1 shows the 17 measures categorized in three dimensions. Two reviewers (RS and JT), who are professors of dental medicine and licensed dentists, independently completed the mapping procedures. A consensus was reached after a discussion of the initial mapping results.

**Table 1:** Composite measures from the Patient Experience Measures from the CAHPS Dental Plan Survey.

Dimensions, items		Composite measures
**Care from dentists and staff**		
	Q6	How often did your regular dentist explain things in a way that was easy to understand?
	Q7	How often did your regular dentist listen carefully to you?
	Q8	How often did your regular dentist treat you with courtesy and respect?
	Q9	How often did your regular dentist spend enough time with you?
	Q11	How often did the dentists or dental staff do everything they could to help you feel as comfortable as possible during your dental work?
	Q12	How often did the dentists or dental staff explain what they were doing while treating you?
**Access to dental care**		
	Q13	How often were your dental appointments as soon as you wanted?
	Q15	If you tried to get an appointment for yourself with a dentist who specializes in a particular type of dental care (such as root canals or gum disease) in the last 12 months, how often did you get an appointment as soon as you wanted?
	Q16	How often did you have to spend more than 15 minutes in the waiting room before you saw someone for your appointment?
	Q17	If you had to spend more than 15 minutes in the waiting room before you saw someone for your appointment, how often did someone tell you why there was a delay or how long the delay would be?
	Q14	If you needed to see a dentist right away because of a dental emergency in the last 12 months, did you get to see a dentist as soon as you wanted?
**Dental plan costs and services**		
	Q19	How often did your dental plan cover all of the services you thought were covered?
	Q22	How often did the 800 number, written materials, or website provide the information you wanted?
	Q27	How often did your dental plan’s customer service give you the information or help you needed?
	Q28	How often did your dental plan’s customer service staff treat you with courtesy and respect?
	Q20	Did your dental plan cover what you and your family needed to get done?
	Q24	Did this information (from your dental plan) help you find a dentist you were happy with?

## Results

### Descriptive Statistics

Table 2 shows the demographics of these dentists. Among the collected data, 154.683 (75.55%) dentists received at least one rating or review. The number of reviews ranged from 1 to 1789. There were more male dentists than females with a percentage difference of 85.87%. The majority of dentists (78.55%) were general dentists, followed by dentists specialized in orthodontics and dentofacial orthopedics (9.23%), pediatrics (4.76%), and endodontics (3.81%). The majority of dentists (91.83%) did not specify any second languages other than English. Among those who indicated speaking a second language, Spanish, Hindi, Arabic, French, and Chinese were most common. There were 150,571 (73.54%) dentists who received an overall rating of ≥3 out of 5, and 50,068 (24.45%) of all dentists received no ratings.

**Table 2:** Dentist demographics.

		Count	Proportion (%)
**Gender**			
	Female	58,309	28.48
	Male	146,044	71.33
	Unknown	398	0.19
**Age**			
	Under 30	1585	0.77
	30-39	28,736	14.03
	40-49	36,715	17.93
	50-59	29,006	14.17
	60-69	29,585	14.45
	70-79	11,716	5.72
	80-89	1826	0.89
	Over 89	157	0.08
	Unknown	65,425	31.95
**Specialty**			
	Dental Anesthesiology	338	0.17
	Endodontics	7803	3.81
	General Dentistry	160,831	78.55
	Oral and Maxillofacial Pathology	166	0.08
	Oral and Maxillofacial Radiology	1	0.00
	Oral and Maxillofacial Surgery	975	0.48
	Orthodontics and Dentofacial Orthopedics	18,891	9.23
	Pediatric Dentistry	9743	4.76
	Periodontics	1541	0.75
	Prosthodontics	4249	2.08
	Other	213	0.10
	Unknown	0	0
**Language**			
	Spanish	3972	1.94
	Hindi	510	0.25
	Arabic	471	0.23
	French	467	0.23
	Chinese	440	0.21
	Russian	429	0.21
	Farsi	338	0.17
	Vietnamese	305	0.15
	Korean	304	0.15
	Portuguese	272	0.13
	Unknown	188,025	91.83
**Rating**			
	1-1.9	1344	0.66
	2-2.9	2768	1.35
	3-3.9	16,431	8.02
	4-4.9	61,520	30.05
	5	72,620	35.47
	Unknown	50,068	24.45
**Wait time**			
	Under 10 minutes	98,104	47.91
	10-15 minutes	46,347	22.64
	16-30 minutes	4395	2.15
	31-45 minutes	880	0.43
	Over 45 minutes	335	0.16
	Unknown	54,690	26.71

### Inferential Statistics

**Figure 2:**
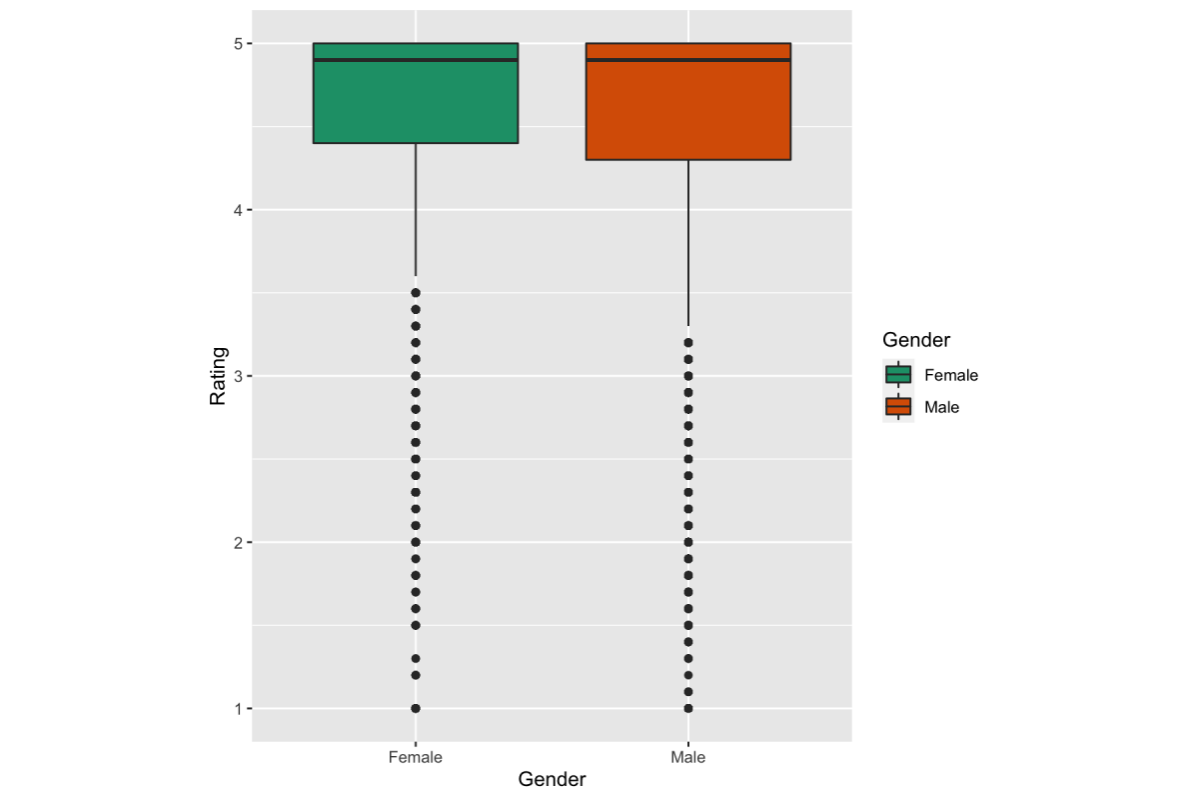
Overall ratings by gender.

ANOVA showed no significant effect of specialties on ratings (*F*_9, 154673_=58.74, *P*=1.37, *g*=0.06). However, the average rating was higher for female dentists (M=4.58) than male dentists (M=4.57) (t_71881_=2.45, *P*<.01, *g*=0.01). See Figure 2.

**Figure 3:**
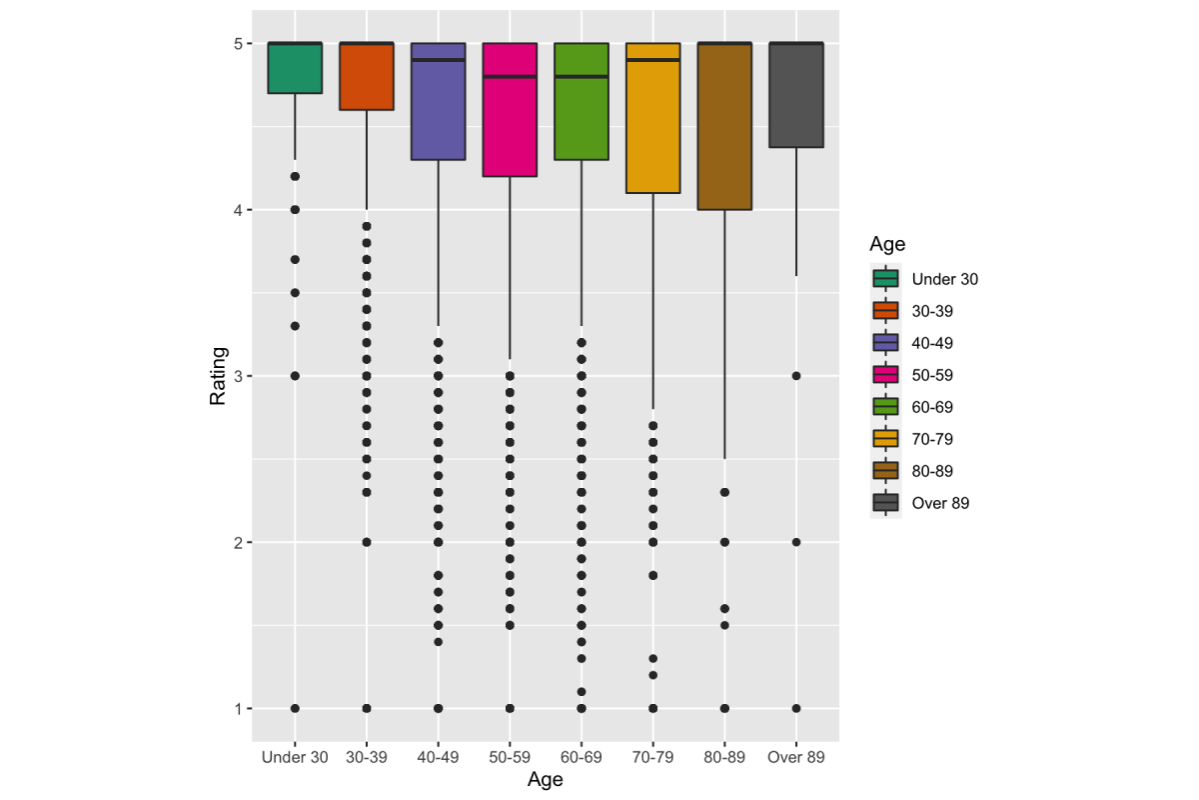
Overall ratings by age group.

We also found a significant effect of age on ratings (*F*_7, 107128_=246.97, *P*<.001, *g*=0.11) with younger age associated with higher ratings. The Tukey Honestly Significant Difference (Tukey HDS) test showed a significant difference in ratings between each pair of age groups except “30-39” vs “Over 89” (*P*=.15, *g*=0.01), “Under 30” vs “30-39” (*P*=.10, *g*=0.01), “40-49” vs “Over 89” (*P*=.95, *g*=0), “50-59” vs “60-69” (*P*=1.00, *g*=0), “50-59” vs “Over 89” (*P*=1.00, *g*=0), “60-69” vs “Over 89” (*P*=1.00, *g*=0), and “80-89” vs “Over 89” (*P*=1.00, *g*=0). See Figure 3.

**Figure 4:**
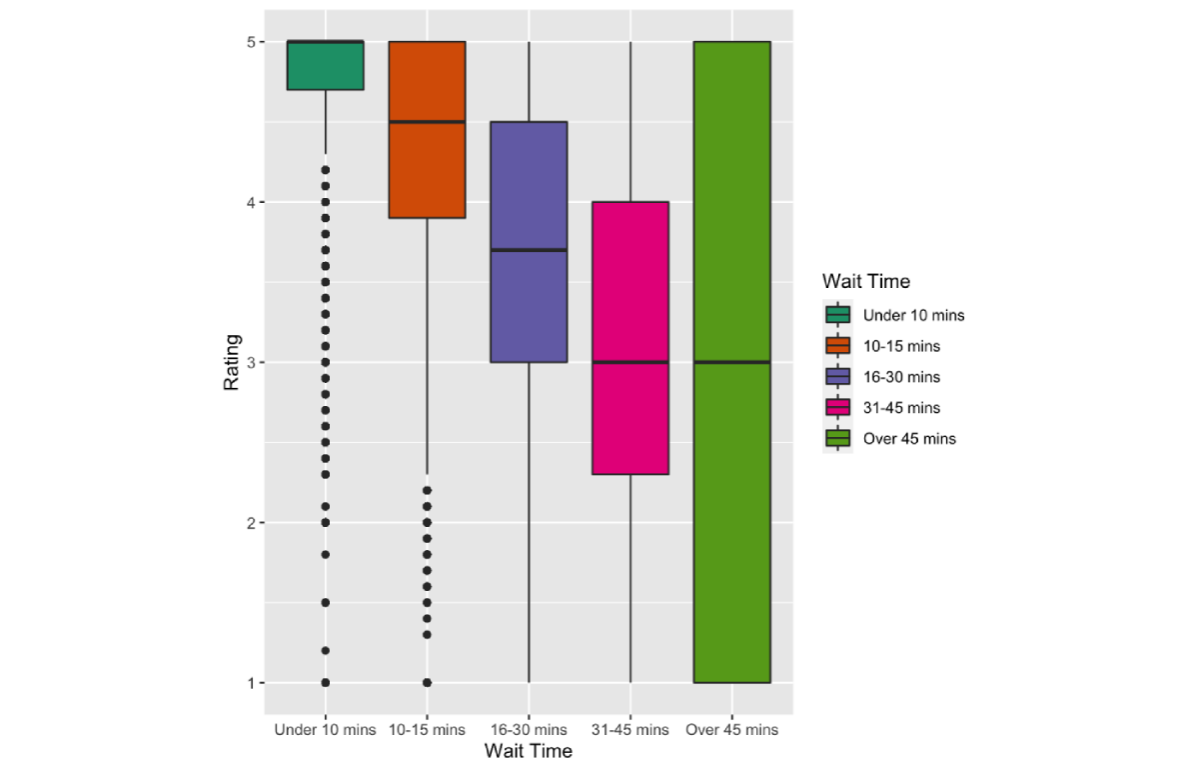
Overall ratings by wait time.

There was also a significant effect of wait time on ratings (F_4, 150055_=10417.77, *P*<.001, *g*=0.26) with shorter wait times associated with higher ratings. Tukey HDS showed statistical significance in comparing the mean difference of ratings in each pair of wait times (all *P*<.001, g=[0.01, 0.18]). See Figure 4.

### Identification of Concepts Relating to Dental Patient Experience

**Figure 5:**
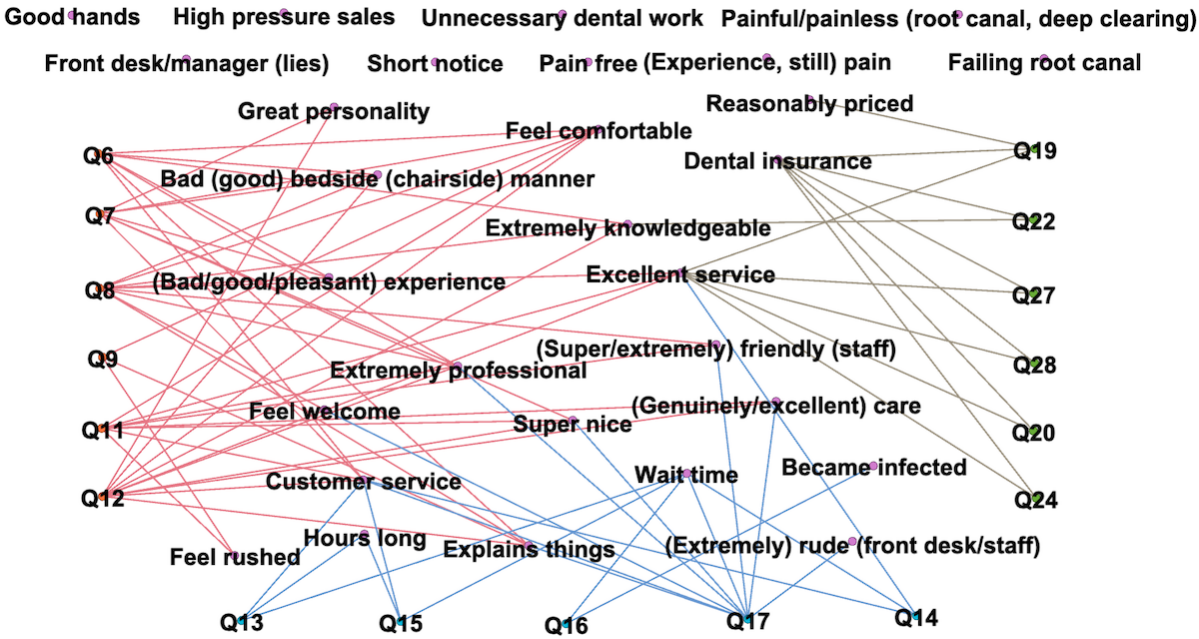
Mapping of Patient Experience Measures from the CAHPS Dental Plan Survey and the words and phrases extracted from patient reviews.

Cohen kappa (equally weighted) was 0.95 between the two raters who independently identified patient experience-related words and phrases from 2000 automatically extracted collocations. After discussion, they identified 29 words and phrases, then two other reviewers of dental experts independently mapped the 29 words and phrases onto the 17 composite measures in the Patient Experience Measures from the CAHPS Dental Plan Survey. Figure 5 shows a map of links. Each composite measure has 2-10 representing words and phrases. Out of the three dimensions of the patient experience, there were more topics representing “care from dentists and staff” and “access to dental care” compared to “dental plan costs and services.” Patients were more likely to discuss their experience with dentists and staff than health insurance providers. There were eight words and phrases related to the patient experience that did not correspond to any composite measures. Some of these topics were specific to dental care. For example, discomfort (eg, painful/painless root canal or deep cleaning) is a common type of feedback from dental patients. Topics relating to ethics (eg, high-pressure sales and unnecessary dental work) have received little attention in dentistry but merits further research.

## Discussion

### Principal Findings

Over the last few years, researchers have begun a systematic analysis of online patient reviews. In the United States, several empirical studies investigating online reviews of general healthcare services and specialties are well documented, but such studies have not been performed in dentistry [1]. To the best of our knowledge, this study represents the first study of online reviews of dental care in the United States. In particular, this study demonstrated that online patient reviews are an essential data source for studying dental patient experience. This study also characterized patient feedback to dental care, which can inform dental care quality improvement.

We found several factors associated with overall dentist ratings. In particular, female dentists were rated slightly higher than their male counterparts, although this should be interpreted with the understanding that the effect size is small (*g*=0.01), and the sample size was small for senior dentists. The gender and age differences we found in this study were similar to a study of online reviews of dentists in Germany [25] as well as reviews of surgeons [26,27], but such a statistical significance was not consistent in other studies. Earlier studies on patient-provider communication suggested that female healthcare providers engage in significantly more active partnership behaviors, positive talk, and building trust with patients [28]. Female providers were also found associated with lower mortality and readmission as compared to their male counterparts [29]. The literature on healthcare quality suggests that younger physicians in acute care hospitals report lower mortality rates [30]. Our data also showed that higher overall ratings were associated with shorter wait times (*g*=0.26). This finding has been reported and discussed in several studies of online patient reviews [1]. Patient review websites such as HealthGrades and RateMDs have included “wait time/punctuality” as a default measure for healthcare providers.

The proliferation of patient review websites represents a wealth of patient-experience data, but these data remain understudied. In this study, we identified unstructured descriptions of patient experience using a method integrating quantitative text mining and qualitative human evaluation. Our method recognizes the role of automated textual data analytics in harnessing information from online reviews, consistent with other recent studies [31-37], although some researchers argued the limitations of text mining because it involves limited supervision by human experts [31]. To minimize the impact of this lack of human oversight, we incorporated human evaluation procedures into the automated natural language processing effort. Our findings showed that patient reviews covered a full range of topics measured by the Patient Experience Measures from the CAHPS Dental Plan Survey and demonstrated a high level of correlation. Among the eight topics not corresponding to any measures from the survey, some topics may have provided a nuanced view of dental care as compared to patient survey responses [36]. These findings suggest that online patient reviews can be used to assess patient experience during dental care.

### Limitations

Our study has the following limitations. First, data from Healthgrades, like data from any other patient review website, may be incomplete and biased. Not all dentist profiles have been claimed. Although authentication is required for dentist profile information, inaccuracies may still exist. The overall ratings may be biased because patients who are happy with health services are more likely to leave ratings and reviews [2]. Second, although there are good reasons for using data from a single review site, this limitation may still weaken the reliability of the study. Repeated measures and follow up studies are needed to evaluate the findings of the this study. Third, it is challenging to differentiate the descriptions of the patient experience from patient satisfaction as patients often write about their satisfactory or unsatisfactory experience with emotions and personal preferences. In this study, we only analyzed words and phrases at a semantic level, while contextual information is limited. Third, text mining is efficient for big data analyses but falls short in domain-specific and context-based analyses, in which traditional qualitative approaches should be considered as an essential complement of text mining [31].

Despite these limitations, this study analyzed an extensive dataset and found an association between dentists and online patient reviews. The thematic analysis also identified themes of patient experience similar to those of CAHPS, suggesting that online patient reviews can inform improved quality in dental care.

### Conclusions

This study demonstrated that PORs are a potential data source that can supply rich performance data from the patient perspective, based on which assessments of dental care quality and the patient experience is feasible. We also identified several factors associated with dentists’ overall ratings, which could be used to inform dental care quality improvement.

## Abbreviations

ADA: American Dental Association
ANOVA: Analysis of Variance
CAHPS: Consumer Assessment of Healthcare Providers and Systems
CMS: Centers for Medicare and Medicaid Services
Tukey HDS: Tukey’s Honestly Significant Difference
